# High-Intensity Interval Training Decreases Muscle Sympathetic Nerve Activity in Men With Essential Hypertension and in Normotensive Controls

**DOI:** 10.3389/fnins.2020.00841

**Published:** 2020-08-18

**Authors:** Thomas Svare Ehlers, Yrsa Sverrisdottir, Jens Bangsbo, Thomas Petursson Gunnarsson

**Affiliations:** ^1^Department of Nutrition, Exercise and Sports, University of Copenhagen, Copenhagen, Denmark; ^2^Nuffield Department of Clinical Neurosciences, Medical Sciences Division, University of Oxford, Oxford, United Kingdom; ^3^Department of Basic Medical Sciences, Mohammed Bin Rashid University of Medicine and Health Sciences, Dubai, United Arab Emirates

**Keywords:** ambulatory blood pressure, sprint interval training, exercise training, blood pressure, 10-20-30 training

## Abstract

Exercise training is a cornerstone in reducing blood pressure (BP) and muscle sympathetic nerve activity (MSNA) in individuals with essential hypertension. High-intensity interval training (HIIT) has been shown to be a time efficient alternative to classical continuous training in lowering BP in essential hypertension, but the effect of HIIT on MSNA levels has never been investigated. Leg MSNA responsiveness to 6 weeks of HIIT was examined in 14 hypertensive men (HYP; age: 62 ± 7 years, night time BP: 136 ± 12/83 ± 8 mmHg, BMI: 28 ± 3 kg/m^2^), and 10 age-matched normotensive controls (NORM; age: 60 ± 8 years, night time BP: 116 ± 2/68 ± 4 mmHg and BMI: 27 ± 3 kg/m^2^). Before training, MSNA levels were not different between HYP and NORM (burst frequency (BF): 41.0 ± 10.3 vs. 33.6 ± 10.6 bursts/min and burst incidence (BI): 67.5 ± 19.7 vs. 64.2 ± 17.0 bursts/100 heart beats, respectively). BF decreased (*P* < 0.05) with training by 13 and 5% in HYP and NORM, respectively, whereas BI decreased by 7% in NORM only, with no difference between groups. Training lowered (*P* < 0.05) night-time mean arterial- and diastolic BP in HYP only (100 ± 8 vs. 97 ± 5, and 82 ± 6 vs. 79 ± 5 mmHg, respectively). The change in HYP was greater (*P* < 0.05) compared to NORM. Training reduced (*P* < 0.05) body mass, visceral fat mass, and fat percentage similarly within- and between groups, with no change in fat free mass. Training increased (*P* < 0.05) V̇O_2_-max in NORM only. Six weeks of HIIT lowered resting MSNA levels in age-matched hyper- and normotensive men, which was paralleled by a significant reduction in BP in the hypertensive men.

## Introduction

Essential hypertension is a multifactorial cardiovascular disease with no simple identifiable cause and a major risk factor for cardiovascular morbidity and mortality worldwide (Carretero and Oparil, 2000; Lewington et al., 2002). The etiology of essential hypertension is complex, but autonomic dysfunction is one mechanism involved in the pathophysiology of essential hypertension (Messerli et al., 2007; Carthy, 2014). Sedentary individuals with essential hypertension have higher resting levels of muscle sympathetic nerve activity (MSNA) compared to normotensive controls (Grassi et al., 1998b, 2015; Smith et al., 2002). This even extends to runners with hypertension (De Sa Perlingeiro et al., 2016) despite that continuous moderate-intensity training (CMIT) is known to lower MSNA levels in hypertensive individuals (Pescatello et al., 2004; Cornelissen and Fagard, 2005; Pedersen and Saltin, 2006). High-intensity interval training (HIIT) has been proposed as a time-efficient and superior alternative to CMIT in lowering blood pressure (BP) in hypertensive individuals and at least as efficient in improving cardio-metabolic risk factors such as the maximum oxygen uptake (V̇O_2_-max) and body composition (Costa et al., 2018). Diet and exercise-induced weight loss has been associated with a decrease in resting MSNA levels (Trombetta et al., 2003; Tonacio et al., 2006; Straznicky et al., 2010, 2011), and exercise-induced weight loss was associated with decreased systolic and diastolic BP (Mehta et al., 2016; Khera et al., 2018). Whether loss of fat mass is a mechanism underlying decreased MSNA and BP levels following a period of HIIT in hypertensive males is unknown.

Only one study has investigated the effects of exercise on BP and MSNA levels in hypertensive individuals (Laterza et al., 2007), albeit other studies have investigated other populations at risk for adverse cardiovascular events, such as patients with chronic heart failure, myocardial infarction, metabolic syndrome, and obesity (Carter and Ray, 2014). In the study by Laterza et al. (2007), never-treated hypertensive men and women had improved baroreflex sensitivity and decreased resting MSNA levels following 4 months of CMIT. The improved baroreflex sensitivity was associated with a decrease in BP in the hypertensive individuals. It is well established that CMIT lowers BP; however, HIIT has been shown to induce health-related benefits equivalent to or greater than CMIT, indicating that exercise intensity may be of importance for reversing key alterations present in the pathophysiology of hypertension and thus for prevention and treatment of hypertension (Ciolac, 2012; Hussain et al., 2016). Nonetheless, the effects of HIIT on resting MSNA levels and the association to alterations in BP and body composition in hypertensive individuals have yet to be investigated. Furthermore, it is unclear how HIIT affects MSNA levels in healthy normotensive individuals (Carter and Ray, 2014).

Compliance is key to obtain the beneficial effects of exercise training, and with lack of time being one of the most cited barriers for not engaging regularly in exercise (Stutts, 2002; Trost et al., 2002; Lidegaard et al., 2016), HIIT may be considered as an attractive exercise modality, as time spent during training may be reduced by up to 50% compared to CMIT (Gunnarsson and Bangsbo, 2012; Gliemann et al., 2015). An emerging HIIT modality is 10-20-30 training, consisting of repeated 10-s sprints followed by 30 and 20 s of low- and moderate-intensity exercise (Gunnarsson and Bangsbo, 2012). Despite the all-out nature of the 10-s sprints, they are wieldy which may be important for long-term adherence in patients with essential hypertension. Recently, we have shown cycling-based 10-20-30 training to be effective in lowering BP and improving vascular function, V̇O_2_-max, and body composition in concert with a high compliance in various patient groups (Fiorenza et al., 2018; Toennesen et al., 2018; Baasch-Skytte et al., 2020; Gunnarsson et al., 2020). It is conceivable that the anti-hypertensive effects of the 10-20-30 training relates to a high adrenergic stressor response and/or marked metabolic perturbations as observed during short-term sprinting (Fiorenza et al., 2018; Gunnarsson et al., 2019), which may reduce the MSNA levels in concert with BP in hypertensive individuals.

In the present study, we tested the hypothesis that 6 weeks of 10-20-30 cycling training would decrease BP in men with essential hypertension and that the decrease in BP would be paralleled by a decrease in resting MSNA levels and fat mass. Furthermore, we hypothesized that the 10-20-30 training would not decrease resting MSNA levels and BP in age-matched normotensive men.

## Materials and Methods

### Ethical Approval

The study was approved by the Scientific Ethical Committee of The Capital Region of Denmark (H-4-2014-100) and conducted in accordance with the guidelines of the Declaration of Helsinki of 2013. The study was registered at ISRCTN.com (ISRCTN11181410).

### Study Participants

Twenty-four sedentary men were included in the study: 14 with essential hypertension [HYP; age: 62 ± 7 years, night-time BP: 136 ± 12/83 ± 8 mmHg, and body mass index (BMI): 28 ± 3 kg/m^2^] and 10 age-matched normotensive controls (NORM; age: 60 ± 8 years, night-time BP: 116 ± 2/68 ± 4 mmHg, and BMI: 27 ± 3 kg/m^2^) (Table 1). Of the 14 hypertensive men, 10 were on antihypertensive medication, including ACE inhibitors, calcium antagonists, diuretics, and beta-blockers (Table 2). All participants were fully informed of the experimental procedures and any discomforts associated with participating in the study before signing a written informed consent.

**TABLE 1:** Anthropometric, health, and performance-related variables before (Pre) and after (Post) 6 weeks of high-intensity cycling training in sedentary hypertensive (HYP, *n* = 14) and normotensive (NORM, *n* = 10) men.

	HYP		NORM	
	Pre	Post	Pre	Post
Age, years	62.1 ± 6.9		59.7 ± 8.0	
Height, cm	175 ± 4^†^		181 ± 8	
Body mass index, kg/m^2^	28.0 ± 3.1	27.5 ± 3.2	26.5 ± 2.9	26.1 ± 2.9
Body mass, kg	85.5 ± 7.7	84.1 ± 7.9*	86.5 ± 12.7	85.3 ± 12.3*
Visceral fat mass, kg	2.0 ± 0.6^†^	1.8 ± 0.7*	1.4 ± 0.7	1.2 ± 0.7*
Fat free mass, kg	54.9 ± 3.7	55.5 ± 4.0	58.9 ± 7.8	59.2 ± 7.4
Resting HR, heartbeats/minute	61.0 ± 9.1^†^	59.3 ± 11.2	51.5 ± 7.4	52.5 ± 7.2
V̇O_2_-max, ml/min	2,860 ± 485	2,871 ± 490	3,214 ± 475	3,397 ± 564^*‡^
GXT time to exhaustion, s	484 ± 118^†^	567 ± 105^*†^	623 ± 160	723 ± 162*
V̇O_2_ during cycling at 50% of V̇O_2_-max, ml/min	1,403 ± 286	1,198 ± 260*	1,627 ± 383	1,494 ± 495*
V̇O_2_ during cycling at 65% of V̇O_2_-max, ml/min	1,858 ± 378	1,622 ± 390^*†^	2,191 ± 460	2,073 ± 516*

Values are mean ± SD. *Different (P < 0.05) from Pre. ^†^Different (P < 0.05) from NORM. ^‡^Group by time interaction (P < 0.05). BF, burst frequency; BI, burst incidence; MAP, mean arterial blood pressure; SYS, systolic blood pressure; DIA, diastolic blood pressure; GXT, graded exercise test; V̇O_2_-max, maximum oxygen uptake.

**TABLE 2:** Medication in 10 of 14 sedentary men with essential hypertension.

1	Bendroflumethiazid/kaliumchlorid 2.5 mg/573 mg
2	Bendroflumethiazid/kaliumchlorid 2.5 mg/573 mg, losartan 100 mg
3	Enalapril 15 mg
4	Enalapril/hydrochlorthiazid 20 mg/12.5 mg, amlodipin, 5 mg
5	Losartan/hydrochlorthiazid 100 + 12.5 mg
6	Losartan/hydrochlorthiazid 100 + 25 mg
7	Losartan/hydrochlorthiazid 50 + 12.5 mg, amlodipin 10 mg
8	Metoprololsuccinat 50 mg, amlodipin 5 mg, bendroflumethiazid/kaliumchlorid 2.5 mg/573 mg
9	Ramipril 10 mg
10	Ramipril/hydrochlorthiazid 5 mg/10 mg, amlodipin 5 mg

### Experimental Design

Prior to enrollment in the study, the subjects underwent a screening procedure. Before and after a 6-week training intervention, the subjects completed two experimental days to evaluate body composition, cardiovascular fitness, and BP (experimental day I) and resting leg MSNA levels (experimental day 2). The experimental days were separated by 48–96 h. The subjects refrained from caffeine, alcohol, and exercise 24 h prior to the experimental days and recorded their food intake in the morning prior to arriving at the laboratory at 8–9 A.M.

### Screening

The screening procedure consisted of six consecutive measurements of clinical BP by an automatic upper-arm BP monitor (M7; OMRON, Vernon Hills, IL, United States), after 20 min of rest in supine position in a quiet dim room, a 12-lead ECG, blood sampling (RBC, Hb, HbA1c, creatinine, CRP, ALAT, ASAT, GGT, APTT, INR, and coagulation factors II + VII + X), a health questionnaire, and a physical evaluation by medically trained personnel. Part of the screening procedure included a graded exercise test (GXT) on a cycle ergometer (50 W for 4 min, followed by an increase in load of 25 W/min until volitional fatigue and/or a drop in cadence below 55 RPM despite strong verbal encouragement) to determine V̇O_2_-max [highest 30-s average with a plateau in V̇O_2_ despite an increase in workload and a respiratory exchange ratio (RER) above 1.10 as objective criteria] using a breath-by-breath gas analyzing system (Oxycon Pro, Viasys Healthcare, Hoechberg, Germany). It should be mentioned that RER values above 1.00 are not indicative of the muscular respiratory quotient as they represent an excess production of CO_2_ by bicarbonate buffering of protons rather than excess muscle substrate utilization. If included in the study, this test served as a habituation trial.

The inclusion criteria were men aged 40–70 years, with a BMI of 20–35 kg/m^2^, clinical BP at screening of >135/85 (hypertensive) or <130/80 mmHg (normotensive), and ambulatory night-time BP of >130/80 mmHg (hypertensive) and <125/75 mmHg (normotensive) (Pickering et al., 2005), engaged in <2 h of physical activity per week, and with a HbA1c level of <6.5% and <48 mmol/mol. The exclusion criteria were chronic diseases other than essential hypertension, use of medication other than anti-hypertensive drugs, and inability to perform physical exercise. No men in HYP were excluded based on the upper limit BP, but the clinical BP of some men in NORM were higher than the guideline values during clinical BP measurements (Pickering et al., 2005). However, all normotensive men had night-time BP values <125/75 mmHg, confirming that these men were in fact normotensive at the onset of the study.

### Experimental Day 1

The subjects abstained from any daily medication for three full days prior to the experimental days in order to reduce the influence of medication during the experimental procedures. A full wash-out of all pharmacological substances would require a longer period, which was not prioritized over subject safety. The subjects reported to the laboratory in the morning after overnight fasting (>10 h), and a whole-body dual X-ray absorption scan (Lunar Prodigy Advance; General Electric, Madison, WI, United States) was completed. At 1 h after a standardized breakfast consisting of two buns (∼150 g) with marmalade (25 g), butter (25 g), cheese (two slices), and juice (∼250 ml), the subjects completed two cycling bouts at 50 and 65% of HR_peak_, respectively (determined from the screening test), separated by 2 min of rest and followed by a GXT to exhaustion to determine V̇O_2_-max and performance (time to exhaustion during the GXT). At 24 h after the experimental day, night-time BP was monitored (TM-2430 PC2, Boso, Jungingen, Germany) in 30-min intervals while the subjects were still abstaining from their medication. The night-time BP measurements were initiated and concluded by the participants when going to bed and upon waking up, respectively. In addition, the subjects were instructed to report working hours, physical activity, including cycling, and meals on the day of the measurement to ensure that pre- and post-measurements were comparable. The night-time BP measurements were completed following a work day, and exclusion of data points was conducted according to standardized criteria: systolic BP > 300 mmHg, systolic BP < diastolic BP, diastolic BP < 40 mmHg, and/or diastolic BP > 130 mmHg. Data analysis during post-testing disclosed that two pre-test VO_2_-max data files (*n* = 2 in HYP) were corrupted and could not be restored, which was why they were excluded from the data set. Furthermore, one pre-test night-time BP data file (*n* = 1 in HYP) was excluded from analysis due to a corrupted data set (insufficient data points).

### Experimental Day 2

**FIGURE 1:**
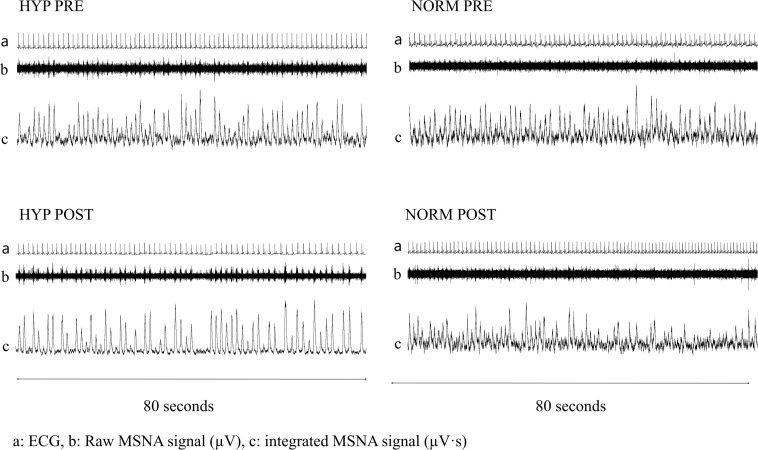
Resting 80 second recordings of ECG (top line; **a**) as well as raw (middle line; **b** μV⋅s) and integrated (bottom line; **c** μV⋅s) muscle sympathetic nerve activity signals before (Pre) and after (Post) 6 weeks of high-intensity cycling training in a representative and sedentary hypertensive (HYP) and normotensive (NORM) men.

The subjects reported to the laboratory at least 2 h after their last meal. MSNA measurements were done *ad modum* Vallbo et al. (1979). The accessibility of the peroneal nerve was assessed by external stimulation (0–10 mA, 1 Hz, and pulse 0.2 ms) with an isolated stimulator (stimulus isolator, ADInstruments). Upon assessing nerve accessibility, an isolated tungsten electrode (FHC, Bowdoin, ME, United States) was inserted into the peroneal nerve posterior to the fibular head. An uninsulated reference electrode was inserted subcutaneously in close proximity (<5 cm). Internal stimulation (0.01–0.1 mA) through the tungsten electrode was conducted in order to place the electrode within a muscle fascicle. Direct recordings of multi-unit efferent post-ganglionic MSNA were then obtained. The raw MSNA signal was amplified (gain 20.000) and filtered (0.3–5 kHz). The MSNA signal was hereafter integrated (absolute integral, time constant decay 0.1 s) in order to improve the visualization of bursts. The bursts were validated by pulse synchronicity, by responsiveness to arousal stimulus (no increase), and by responsiveness to inspiratory apnea (increase). The MSNA signal was recorded (10 kHz data points; Powerlab 8/16, Labchart 8 software, ADinstruments, Sydney, Australia) and stored for later analysis. Resting measurements were recorded over a 5-min period preceded by at least 30 min of rest in the supine position. The representative recordings of MSNA are depicted in Figure 1. During post-testing, we were unable to acquire MSNA signals in two subjects (*n* = 1 in HYP and *n* = 1 in NORM.

### Exercise Training Intervention

The training intervention lasted for 6 weeks and consisted of 10-20-30 training (Gunnarsson and Bangsbo, 2012) conducted as cycling to minimize the risk of injury. The subjects completed two weekly training sessions in the first 2 weeks, increasing to three weekly training sessions from week 3. The intervention period was prolonged by one training session for every 3 days until the subjects had completed post-testing. The subjects in HYP and NORM completed a similar amount of training sessions during the training intervention period (Table 3). Each training session consisted of 5–10 min of low-intensity cycling (30–50 W) as warm-up followed by 2 × 5 min (weeks 1 and 2) or 3 × 5 min (weeks 3 to 6) of the following regime: 30 s of low-intensity cycling (30–80 W), 20 s of moderate-intensity cycling (50–120 W), and 10 s of maximal sprint efforts (>300 W). The 5-min exercise bouts were separated by 3 min of passive rest. All training sessions were supervised, and heart rate was monitored (Polar team 2 system, Polar, Electro Oy, Kempele, Finland).

**TABLE 3:** Training characteristics in hypertensive (HYP, *n* = 14) and normotensive (NORM, *n* = 10) men.

Group	Training sessions, number	Total training time (% of HR_*max*_), %					
		<70	71–80	81–85	86–90	91–95	96–100
HYP	18.3 ± 2.0	19 ± 8	30 ± 10^†^	24 ± 8^†^	19 ± 9^†^	7 ± 8^†^	1 ± 2
NORM	17.0 ± 1.2	16 ± 6	18 ± 3	19 ± 7	28 ± 5	17 ± 11	2 ± 3

*Values are mean ± SD. ^†^Different (P < 0.05) from NORM.*

### Data Analysis

The resting MSNA levels were calculated from 5-min recordings with the following criteria: signal-to-noise ratio >2:1, no baseline shift, and constant delay of signal from ECG R-spike. The MSNA levels were reported as burst frequency (BF; number of bursts per minute) and burst incidence (BI; number of bursts per 100 heartbeats). The *post hoc* analysis of MSNA recordings was standardized by the use of Matlab (MathWorks, Natick, MA, United States). In addition, all files were manually inspected to verify that no artifacts were mistaken for bursts. The analysis of MSNA recordings was performed by an expert-level researcher with several decades of experience within the field.

### Statistics

The analysis of between-groups differences at baseline was based on *n* = 14 and *n* = 10 in HYP and NORM, respectively, for all parameters except for night-time BP (*n* = 13) and V̇O_2_-max (*n* = 12) in HYP. The analysis of within-group changes in MSNA, night-time BP, and V̇O_2_-max in HYP was based on *n* = 13, *n* = 13, and *n* = 12, respectively, whereas the analysis of within-group changes in MSNA levels was based on *n* = 9 in NORM. Between-groups differences at baseline and after the training intervention were determined using a linear mixed model with group (HYP, NORM) and time (Pre, Post) as fixed factors and subject as random factor. To estimate the effect of the training intervention within groups, a linear mixed model with time as fixed factor and subjects as random factor was deployed. In addition, between-groups differences in the training-induced change across time were estimated by a linear mixed model with group–time interaction as fixed factor and subject as random factor. To limit the confounding influence of factors other than essential hypertension in the statistical analysis of MSNA levels and BP, covariates were included in the model (age, baseline V̇O_2_-max, and baseline body fat percentage). The baseline body fat percentage was omitted as a covariate for measures of body composition (weight, fat percentage, visceral fat mass, and fat-free mass) and performance (V̇O_2_ during cycling at 50 and 65% of V̇O_2_-max and time to exhaustion during GXT), and baseline V̇O_2_-max was omitted as a covariate in the analysis of V̇O_2_-max.

To compare training load between groups across training time spent in heart rate zones (<70, 71–80, 81–85, 86–90, 91–95, and >95% of maximal heart rate), a linear mixed model with group–time in heart rate zone interaction as fixed factor and subjects as random factor, with age and baseline V̇O_2_-max as covariates, was used. Model checking was based on Shapiro Wilk’s test and *Q*–*Q* plots. In case of heteroscedasticity, i.e., unequal variance, log transformation was applied prior to analysis. Model-based *t*-tests were used in pairwise comparisons to identify between-groups and within-group differences. The level of significance for all analyses was defined as α > 0.80 and *P* < 0.05. Statistical analyses were carried out in R ver. 3.4.1, including the extension packages “lme4” and “multcomp.” The data are presented as mean values with standard deviation (±SD) in the tables and as both mean and individual values in the figures, unless stated otherwise.

## Results

### Muscle Sympathetic Nerve Activity and Resting Heart Rate

**FIGURE 2:**
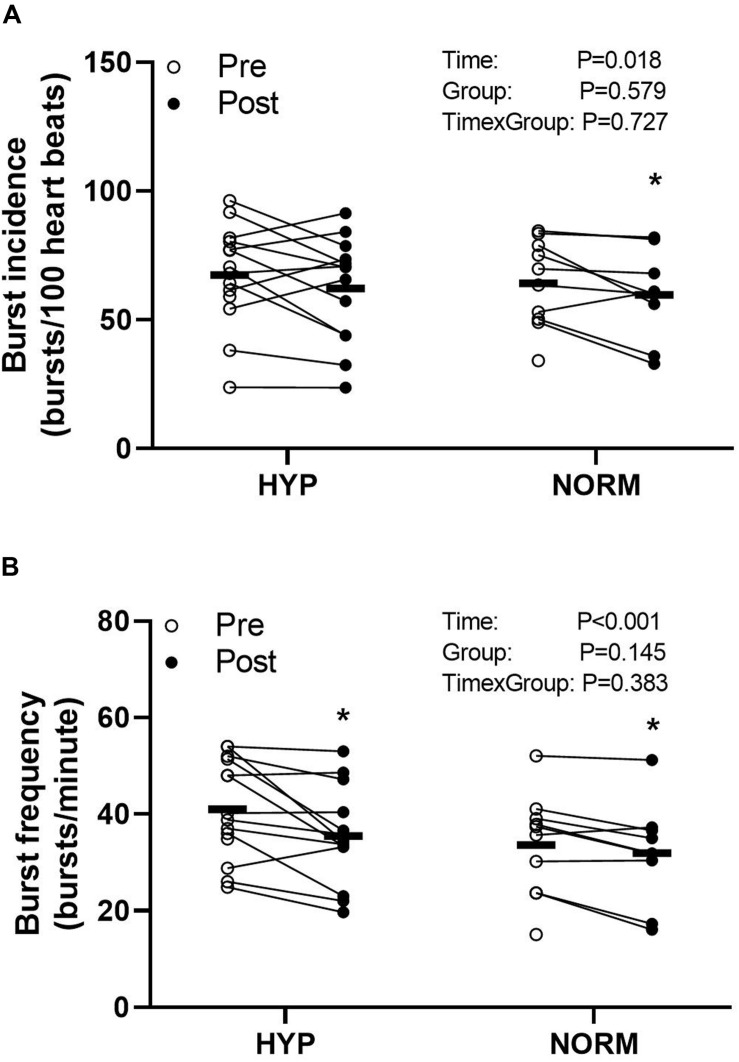
Burst incidence (bursts per 100 heartbeats) **(A)** and burst frequency (bursts per minute) **(B)** before (Pre) and after (Post) 6 weeks of high-intensity cycling training in sedentary hypertensive (HYP, *n* = 14) and normotensive (NORM, *n* = 10) men. *Different (*P* < 0.05) from Pre.

Before the training intervention, the resting MSNA levels were not different between the groups (Figure 2). In HYP, training lowered (*P* < 0.05) the resting MSNA levels, expressed as BF and BI, by 6 ± 7 bursts/min and non-significantly by 6 ± 13 bursts/100 heartbeats, respectively, and by 8 ± 9 bursts/min and 4 ± 3 bursts/100 heartbeats, respectively, in NORM, with no between-group differences (Figure 2). No change in resting heart rate was observed with the training intervention in either group (Table 1).

### Blood Pressure

**FIGURE 3:**
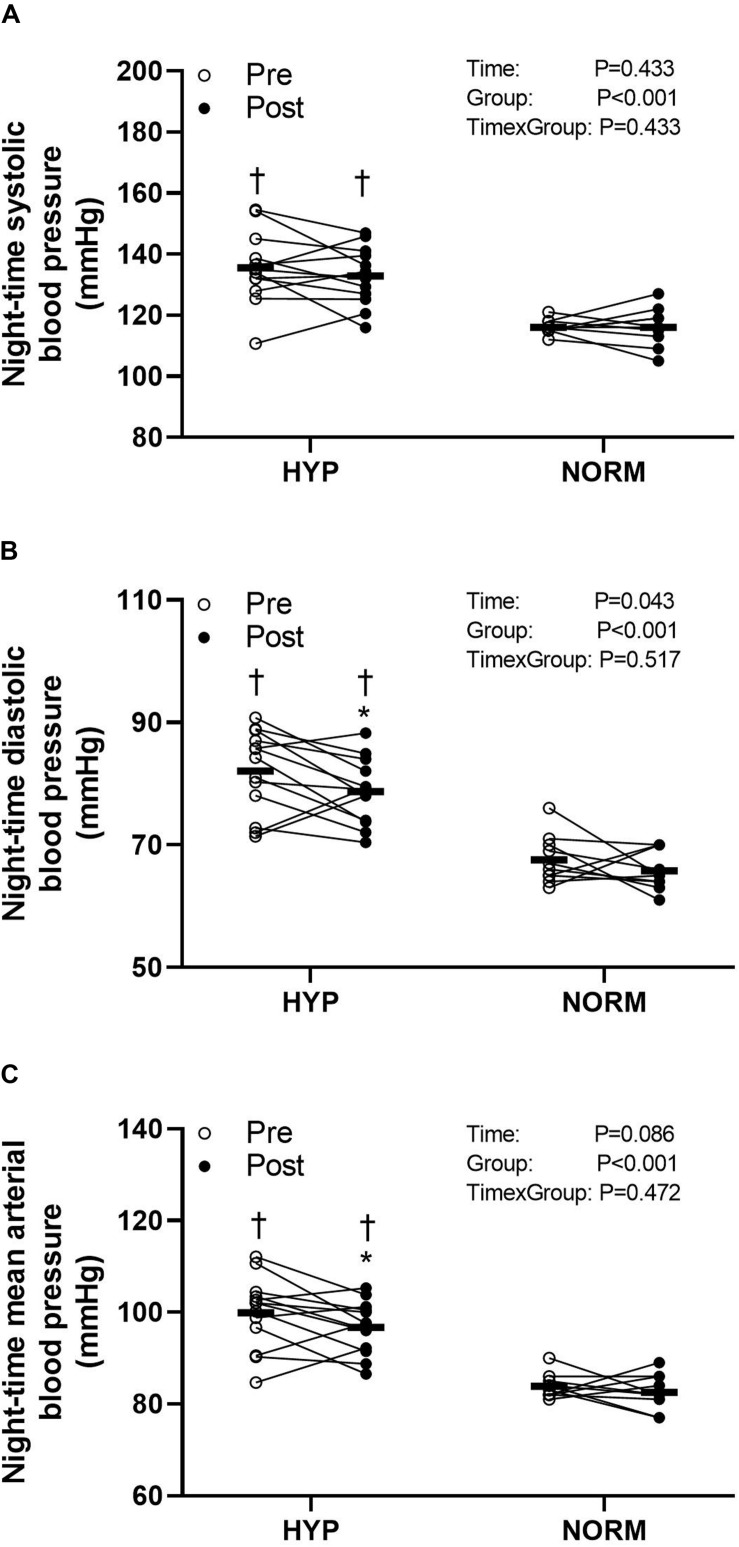
Night-time systolic **(A)** and diastolic **(B)** and mean arterial **(C)** blood pressure before (Pre) and after (Post) 6 weeks of high-intensity cycling training in sedentary hypertensive (HYP, *n* = 14) and normotensive (NORM, *n* = 10) men. *Different (*P* < 0.05) from Pre. ^†^Different (*P* < 0.05) from NORM.

Before the training intervention, the night-time systolic, diastolic, and mean arterial BP was higher (*P* < 0.05) in HYP compared to NORM (higher by 20, 15, and 17 mmHg, respectively) (Figure 3). In HYP, training lowered (*P* < 0.05) the night-time diastolic and mean arterial BP by 4.0 ± 6.3 and 3.5 ± 6.9 mmHg, respectively, with no change in night-time systolic BP (Figure 3). No change in BP was observed with the training intervention in NORM (Figure 3).

### Anthropometrics

**FIGURE 4:**
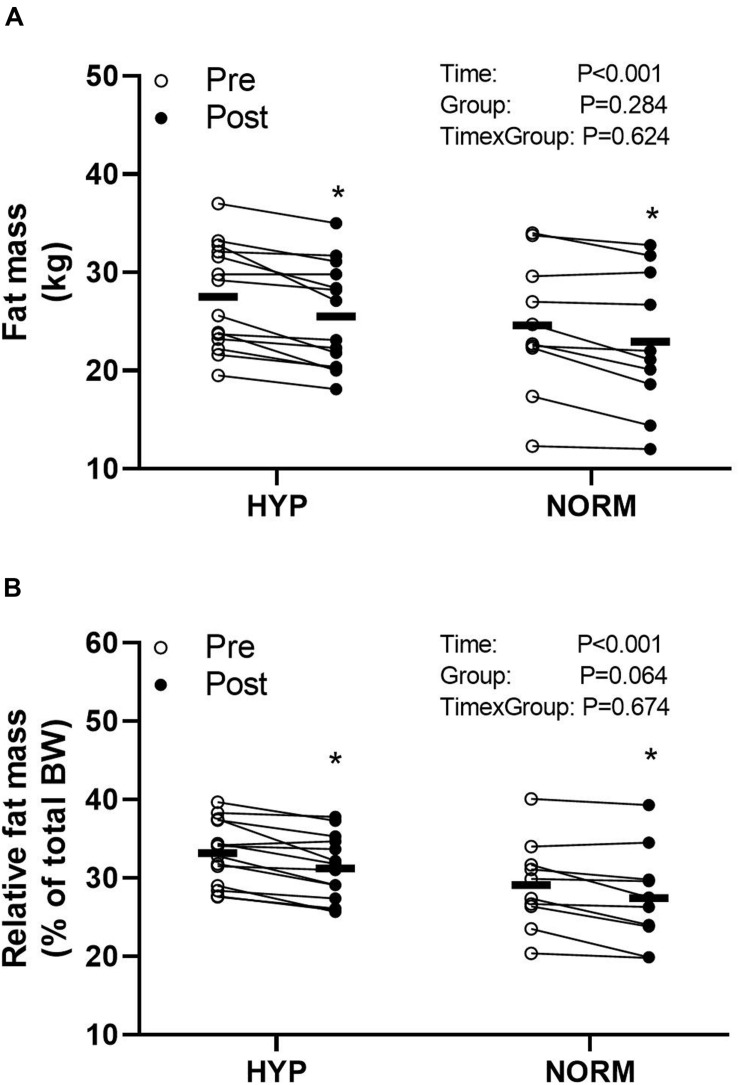
Fat mass **(A)** and relative fat mass **(B)** before (Pre) and after (Post) 6 weeks of high-intensity cycling training in sedentary hypertensive (HYP, *n* = 14) and normotensive (NORM, *n* = 10) men. *Different (*P* < 0.05) from Pre.

Before the training intervention, visceral fat mass was higher (*P* < 0.05) in HYP compared to NORM, whereas no between-groups differences were observed for weight, fat percentage, and fat-free mass (Table 1 and Figure 4). Training reduced (*P* < 0.05) the weight, visceral fat mass, and fat percentage similarly within and between groups, whereas fat-free mass did not change within or between groups (Table 1 and Figure 4).

### Pulmonary Oxygen Uptake and Performance

Before the training intervention, performance during a GXT was lower (*P* < 0.05) in HYP than in NORM, whereas V̇O_2_-max was not different between groups (Table 1). Training increased (*P* < 0.05) V̇O_2_-max only in NORM, and the training-induced change was greater (*P* < 0.05) than in HYP. Training improved (*P* < 0.01) performance during a GXT similarly within and between groups. Performance after the training intervention was lower (*P* < 0.05) in HYP than in NORM.

Pulmonary V̇O_2_ during cycling, at an intensity corresponding to 50 and 65% of V̇O_2_-max, was similar between groups before the training intervention. With training, pulmonary V̇O_2_ decreased (*P* < 0.01) similarly in HYP and NORM (Table 1). The training-induced decrease in V̇O_2_ during cycling at 65% of V̇O_2_-max was greater (*P* < 0.05) in HYP than in NORM, and after the training intervention, the pulmonary V̇O_2_ during cycling at 65% of V̇O_2_-max was lower (*P* < 0.05) in HYP than in NORM (Table 1).

## Discussion

The main findings of the present study were that 6 weeks of 10-20-30 cycling training lowered the MSNA levels similarly between age-matched hypertensive and normotensive men. In addition, the decrease in resting MSNA levels was associated with a decrease in BP in the hypertensive men only. Furthermore, the resting MSNA levels were not different between groups at baseline despite the difference in hypertension status.

The baseline MSNA levels of the present study were similar to observations in hypertensive individuals with similar age (Yamada et al., 1989; Grassi et al., 1998a, b; Spaggiari et al., 2019), but higher than in younger, never-treated individuals with familiar hypertension (Laterza et al., 2007). Despite the relatively short intervention period, the resting MSNA levels decreased in both hypertensive and normotensive men, which likely relates to the higher intensity training in the present study, including repeated 10-s maximal sprints. This type of HIIT would induce a higher hormetic response, including near-maximal heart rates (>90% of HR_max_), a higher adrenergic response (Brandt et al., 2016; Gunnarsson et al., 2019), and muscle fiber recruitment (Krustrup et al., 2004), with greater metabolic perturbations of the contracting muscles (Brandt et al., 2016; Fiorenza et al., 2018; Gunnarsson et al., 2019) compared to lower-intensity continuous training.

Recently, HIIT was suggested to be superior to CMIT in improving markers of sympathetic activity in both hypertensive and normotensive individuals at high familial risk for hypertension (Ciolac, 2012). However, the training-induced reductions in MSNA levels in the present study were lower (6 vs. 12 bursts/min and 5 vs. 20 bursts/100 heartbeats) compared to the changes in hypertensive individuals following CMIT (4 months of 3 × 40 min of exercise training at 70% of V̇O_2_-max per week) (Laterza et al., 2007). The inter-study discrepancies in the training-induced changes in resting MSNA levels may relate to age (61 vs. 46 years), exercise duration (6 weeks vs. 4 months), and/or the use of medication in the present study, where 10 of the 14 hypertensive men were treated with one or more antihypertensive medications. When comparing the results of the medicated and the non-medicated men in the present study, no difference in MSNA levels in response to the 10-20-30 training was observed, albeit these comparisons must be interpreted with caution due to the low numbers.

The underlying mechanisms for the reduced resting MSNA levels in the hypertensive men in the present study are not clear, but increased baroreflex sensitivity may be one explanation as it has been shown to improve in concert with a decrease in resting MSNA levels following 4 months of CMIT in never-treated hypertensive individuals (Laterza et al., 2007). Another possible explanation for the reduction in MSNA levels is the 2.0-kg reduction in fat mass in the hypertensive men as loss of fat mass is associated with a decrease in resting MSNA levels (Trombetta et al., 2003; Tonacio et al., 2006; Straznicky et al., 2010, 2011). In addition, fat mass decreased by 1.7 kg in the normotensive group, which was also associated with a decrease in resting MSNA levels. However, it should be mentioned that, in studies associating reductions in MSNA with loss of body fat (Trombetta et al., 2003; Tonacio et al., 2006; Straznicky et al., 2010, 2011), fat loss was more than 4 kg, and to what extent the average 1.7–2.0 kg loss of body fat in the present study can explain the observed reductions in MSNA is unclear. It should be mentioned that no association between decrease in resting MSNA levels (BI and BF) and reduction in body fat (*r* = 0.16 and *r* = 0.06; *P* > 0.05, respectively) and visceral fat mass (*r* = 0.01 and *r* = 0.29; *P* > 0.05, respectively) as well as in the decrease in night-time systolic (*r* = 0.14 and *r* = 0.01, respectively) and diastolic (*r* = 0.02 and *r* = 0.15; *P* > 0.05, respectively) BP was observed across the hypertensive and the normotensive men of the present study. Whether the lack of association between decrease in MSNA and reduction in body composition and night-time BP can be explained by the limited sample size of the present study is unclear.

The MSNA levels of the normotensive group were similar to the levels of the hypertensive group at baseline and similar to those of normotensive individuals of a similar age (Ng et al., 1993; Hart et al., 2009) but higher than normotensive individuals of lesser age (<50 years) (Ng et al., 1993; Grassi et al., 1998a; Smith et al., 2002; Notarius et al., 2015). The present study is the first to show that 6 weeks of intense HIIT can lower the resting MSNA levels in normotensive men, which is in contrast to a number of studies showing no change in MSNA levels with training in healthy adults (Carter and Ray, 2014). It may be related to the fact that the normotensive men in the present study had high initial resting MSNA levels and several of the same risk factors as the hypertensive individuals by being sedentary and overweight or to the intense nature of the 10-20-30 training intervention. Previous studies comparing the resting MSNA levels in normotensive controls and in patients with essential hypertension have rendered opposing results, showing both similar and higher MSNA levels in the hypertensive state (Yamada et al., 1989; Gudbjörnsdoìttir et al., 1996; Schobel et al., 1996; Notarius et al., 2015). While there is a well-established inverse intra-individual relationship between MSNA and spontaneous BP variations, no clear inter-individual relationship exists between resting levels of MSNA and BP levels in normotensive individuals (Sundlöf and Wallin, 1978). However, an association between MSNA and BP has been observed with aging and in pathological conditions such as secondary hypertension and obesity (Yamada et al., 1989; Grassi et al., 1998a, b; Hart et al., 2009, 2012). In hypertensive individuals, similar to those in the present study, the resting MSNA levels were higher than in the normal population (Yamada et al., 1989; Grassi et al., 1998b; Smith et al., 2002), supporting a positive correlation between resting MSNA levels and BP in elderly individuals (Hart et al., 2012). The observed changes in resting MSNA levels in the hypertensive men were accompanied by a reduction in night-time diastolic and mean arterial BP of 3 and 3 mmHg, respectively, supporting a relation between change in MSNA and change in BP in the hypertensive state. On the other hand, no change in BP was observed in normotensive men, despite reductions in MSNA levels, suggesting that no association between change in MSNA and BP regulation exists in the normotensive state despite their status as sedentary and overweight.

A positive effect of training on resting BP is well established (Hussain et al., 2016), but the importance of duration and intensity of training is unclear. CMIT performed for 30–40 min, two to three times weekly for 10–12 weeks, decreased systolic and diastolic BP by 6–8 and 5–6 mmHg, respectively (Wasfy and Baggish, 2016; Costa et al., 2018). Similarly, HIIT conducted as 4 × 4-min intervals at an intensity of 85–90% of HR_max_ three times weekly for 10–16 weeks decreased the BP by >5 mmHg in hypertensive individuals (Costa et al., 2018). In the present study, decreases in diastolic and systolic BP were observed in the hypertensive men despite a much shorter training intervention (6 weeks), training two- to three times weekly (total of 18.3 training sessions). Each training session lasted less than 25 min, of which only 100–150 s was conducted as sprinting, the remainder was spent at low- to moderate-intensity training during or rest between bouts. Thus, the effect of 6 weeks of 10-20-30 cycling training was comparable to that obtained with longer periods of CMIT and traditional HIIT, i.e., 4 × 4-min intervals in terms of lowering BP and MSNA levels in hypertensive men.

It is notable that the hypertensive group did not improve their V̇O_2_-max during the training intervention, which is in contrast to the normotensive group and to previous observations in similar patient groups implementing 10-20-30 cycling training (Fiorenza et al., 2019; Gunnarsson et al., 2020). That V̇O_2_-max did not improve with training in the hypertensive individuals of the present study is not easily explained as 7–8 weeks of 10-20-30 training improved the V̇O_2_-max in trained runners (Gunnarsson and Bangsbo, 2012; Gliemann et al., 2015). A possible explanation for the lack of improvement in V̇O_2_-max in the hypertensive men is that they spent less of total training time, >85% of HR_max_, compared to the normotensive group, which could relate to the pathology of hypertension and/or the use of medication. Furthermore, the similar change between groups with training in metabolism, i.e., VO_2_ during submaximal cycling and performance during the GXT, could reflect that the training-induced improvements in the skeletal muscle of the hypertensive men in the present study is unaffected by the use of medication. Our data may suggest that the use of medication in the hypertensive men may negatively affect training-induced changes in the cardio-pulmonary system, i.e., in VO_2_-max, but not peripheral training-induced adaptations related to aerobic and anaerobic metabolism.

The safety of training in individuals with essential hypertension should be considered as exercise training induces a marked load on the heart and elevates BP (Glezer and Lediashova, 1975; De Champlain et al., 1991; Lund-Johansen, 1991; Goodman et al., 1992). Recent reviews estimated the risk of sudden cardiac events and found that individuals undergoing supervised programs lowered their risk of sudden cardiac events (Thomas et al., 2011; Millar and Goodman, 2014). HIIT has been suggested to be safe to use even for high-risk populations, such as in essential hypertension, with no reports of adverse events (Weston et al., 2014). This is in line with experiences at our lab conducting HIIT studies for the past 10 years in high-risk patient groups with no adverse events. Furthermore, HIIT, conducted in a group setting, has been reported to be more enjoyable and to improve the quality of life more than CMIT (Wisloff et al., 2007; Ottesen et al., 2010; Molmen-Hansen et al., 2012; Fu et al., 2013), and the constant change of pace during the 10-20-30 training has been reported as motivating by subjects in the present and the previous studies from our lab using this training modality. The latter may be of great importance as motivation is key for adherence to exercise training. Taken together, we suggest HIIT, conducted as 10-20-30 cycling training, to be a time-efficient alternative to classical CMIT and easily applicable for high-risk patient groups, with beneficial effects on MSNA levels and overall health profile in both hypertensive and normotensive men.

## Study Limitations

The present study only included men, which reduces the generalizability of study outcomes. Moreover, the study was conducted in a limited sample size, which did not negatively affect the primary study outcome as the resting MSNA levels were reduced with the intense training intervention in both the hypertensive and the normotensive men.

## Perspectives

The studies investigating the effect of training in at-risk populations have mainly used longer periods of training, whereas the current study suggests that a short period of motivating HIIT, conducted as 10-20-30 cycling training, improves MSNA in essential hypertension. Future studies should provide insight into when and how these adaptations in the nerve system occur in order to further improve our understanding of which type of exercise training is most likely beneficial as a prevention and treatment strategy against hypertension in different cardio-metabolic patient groups.

## Disclosure

JB and TG have authored a book on performance and health benefits of 10-20-30 running training in healthy individuals.

## Data Availability Statement

The raw data supporting the conclusions of this article will be made available by the authors, without undue reservation.

## Ethics Statement

The studies involving human participants were reviewed and approved by the Scientific Ethical Committee of The Capital Region of Denmark. The patients/participants provided their written informed consent to participate in this study.

## Author Contributions

TE was medical responsible on the project. TE, JB, and TG conceived and designed the study. TE, YS, and TG performed the experiments and contributed to data analysis. TE, YS, JB, and TG interpreted the results. TE and TG drafted the manuscript. YS and JB edited and revised the manuscript for important intellectual content. All authors approved final version of the manuscript and agrees to be accountable for all aspects of the work in ensuring that questions related to the accuracy or integrity of any part of the work are appropriately investigated and resolved.

## Conflict of Interest

The authors declare that the research was conducted in the absence of any commercial or financial relationships that could be construed as a potential conflict of interest.
